# The value of surgical admissions for malignant uterine cancer. A comparative analysis of robotic, laparoscopic, and laparotomy surgery in a university hospital

**DOI:** 10.3389/fpubh.2022.920578

**Published:** 2022-10-06

**Authors:** Maria Lucia Specchia, Giovanni Arcuri, Andrea Di Pilla, Emanuele La Gatta, Tommaso Osti, Prospero Limongelli, Giovanni Scambia, Rocco Domenico Alfonso Bellantone

**Affiliations:** ^1^Clinical Governance Department, Fondazione Policlinico Universitario A. Gemelli IRCCS, Rome, Italy; ^2^Life Sciences and Public Health Department, Faculty of Medicine, Università Cattolica del Sacro Cuore, Rome, Italy; ^3^Health Technologies and Innovation Department, Fondazione Policlinico Universitario A. Gemelli IRCCS, Rome, Italy; ^4^Health Department, Fondazione Policlinico Universitario A. Gemelli IRCCS, Rome, Italy; ^5^Woman, Child and Public Health Department, Fondazione Policlinico Universitario A. Gemelli, IRCCS, Rome, Italy; ^6^Translational Medicine and Surgery Department, Faculty of Medicine, Università Cattolica del Sacro Cuore, Rome, Italy

**Keywords:** value-based healthcare, oncology, public health, healthcare system, robotic

## Abstract

**Background:**

Robotic surgery for malignant uterine cancer raises issue of economic sustainability for providers. The objective of this study was to assess the value of surgical admissions for malignant uterine cancer in a University Hospital through an analysis of their costs and outcomes by comparing three different surgical approaches (laparotomy, laparoscopic, and robotic surgery).

**Methods:**

Hospitalizations between 1 January 2019 and 31 October 2021 for malignant uterine cancer surgery were selected and stratified. For each surgical approach, mean values (with 95% confidence intervals, CI) were calculated for cost items. Moreover, 30-day readmission frequency was calculated for the three approaches compared to each other. ANOVA and Student's *t*-test and relative risk (RR) were used for statistical analysis. A break-even analysis was carried out by evaluating the volume of robotic and non-robotic surgical admissions.

**Results:**

A total of 1,336 hospitalizations were included in the study, 366 with robotic, 591 with laparoscopic, and 379 with laparotomy surgery. Robotic surgery, compared to laparoscopic and laparotomy ones, showed a statistically significant difference *(p* < *0.001)* in the economic margin, which was largely negative (−1069.18 €; 95%CI:−1240.44-−897.92 €) mainly due to devices cost, and a lower percentage of 30-day readmissions (1.4%; 95%CI: 0.2–2.6%), with a statistically significant difference only vs. laparotomy *(p* = *0.029)*. Laparoscopic compared to laparotomy surgery showed a significantly *(p* < *0,001)* more profitable economic margin (1692.21 €; 95%CI: 1531.75 €−1852.66 €) without a significant difference for 30-day readmissions. Break-even analysis showed that, on average, for each malignant uterine cancer elective surgery performed laparoscopically, 1.58 elective robotic surgeries are sustainable for the hospital (95% CI: 1.23–2.06).

**Conclusion:**

Break-even analysis could be a useful tool to support hospital management in planning and governance of malignant uterine cancer surgery. Systematic application of this tool will allow defining over time right distribution of robotic, laparoscopic, and laparotomy surgeries' volumes to perform to ensure both quality and economic-financial balance and therefore value of uterine oncological surgery. Concerning research, this study paves the way for a multicentric study, the extension of outcomes of malignant uterine surgery to be considered and assessed, and the future inclusion of other therapeutic interventions in the analysis.

## Introduction

In the early 1990s, the need to move away from a purely volume-driven system in favor of a more value-driven one began to emerge in the area of health services management. This meant focusing more on the quality of care than its volume (1).

The focus on value-driven healthcare increased in 2006 when Porter and Teisberg introduced the concept of value-based healthcare (VBHC), a new strategy for delivering and measuring healthcare (2–4).

The constitutive element of the VBHC concept is that value is defined as the measured improvement in a patient's health outcomes for the cost of achieving that improvement. The value can be increased by lowering healthcare costs or improving care outcomes, or both (1).

The foundational element of VBHC is the concept of measurement: On the one hand, the ultimate goal of healthcare is to improve the health status of the patient, but on the other hand, it is necessary to stay within certain spending limits. Therefore, in a value-based analysis, it is essential to measure both outcomes and costs of individual patient care processes. The results from these analyses allow us to understand whether they are doing well and where to improve in terms of care and efficiency (5).

This approach has found widespread success in modern healthcare management (1), and value dimensions are widely represented among the performance dimensions in hospital care (6).

The value-performance approach can find effective application in oncological surgery of malignant neoplasms of the uterus whose costs and outcomes have been reported in the scientific literature (7–10).

Indeed, endometrial cancer for instance is the most common gynecologic malignancy in developed countries and among most frequent women's cancers, with 8300 estimated cases in 2020 and 3100 estimated deaths in 2021 in Italy. Cervical cancer also plays an important role in terms of disease burden with 2400 cases in 2020 among women in Italy (11, 12).

These simple epidemiological data make uterine neoplastic diseases a focal element on which to concentrate modern therapeutic efforts (13, 14).

The standard treatment of endometrial cancer is laparoscopic hysterectomy with bilateral adnexectomy and pelvic and para-aortic lymphadenectomy or sentinel lymph node evaluation, which, in specialized centers, has replaced lymphadenectomy. In cases where the laparoscopic approach is not feasible, a laparotomy is performed (11).

Most cervical cancers are diagnosed at an early stage and are amenable to surgical management (15).

Abdominal radical hysterectomy, along with the standard surgical management approach for early-stage cervical cancer, achieves excellent survival outcomes. As an alternative minimally invasive surgery to abdominal radical hysterectomy, laparoscopic radical hysterectomy has been used since the early 1990s (16).

The better perioperative outcomes of laparoscopic than abdominal radical hysterectomy are well accepted, despite a lack of well-designed prospective randomized controlled trials. Compared with abdominal radical hysterectomy, laparoscopic one is associated with less estimated blood loss, reduced transfusion requirement, a shorter hospital stay, and less postoperative complications (17).

The gynecologic surgery scenario changed substantially in 2005 with the approval of the use of robotic surgery. Since then, robotic radical hysterectomy and robotic radical trachelectomy have increasingly been used in the surgical treatment of early-stage cervical cancer (18).

In the last 10 years, the offer of minimally invasive therapies, and in particular robotic surgery, has increased in the treatment of uterine cancer, to the detriment of laparotomic surgery (19).

Compared with conventional laparoscopic surgery, robotic surgery platforms have several advantages, including improved instrument dexterity, higher degrees of freedom for instrument movement, a three-dimensional view with a higher magnification, and filtered tremor (20).

Even as far as outcomes are concerned, robotic surgery applied to uterine cancer seems to be better than laparoscopic in terms of hospital stay, return to normal activity, return to a normal diet, conversions to laparotomy, operative complications, blood loss, and overall complications (21).

It is therefore understandable that the resonance of this approach is rapidly increasing, not least because of the short learning curve related to the technology use (22, 23).

Robotic surgery is, on the contrary, characterized by high initial purchase costs of the technology and additional maintenance and surgical costs, the latter higher than those of the laparoscopic technique (12, 19).

In particular, the greatest proportion of robotic hysterectomy costs seems to be associated with time spent in the operating room (24).

Based on the available scientific evidence, we can therefore state that robotic surgery has a strong potential for improving outcomes for patients with malignant neoplasms of the uterus, but at high costs when compared with laparoscopic and laparotomic approaches. The objectives of our study are (a) to assess the value of surgical admissions for malignant uterine cancer in a University Hospital through an analysis of their costs, revenues and outcomes by comparing laparotomy, laparoscopic, and robotic surgery and (b) to assess the economic sustainability of robotic procedures in the same context.

## Materials and methods

This study is compliant with the Local Ethical Committee Standards of the Fondazione Policlinico Universitario Agostino Gemelli (FPG) Scientific Research and Care Institute (IRCCS). It was carried out in accordance with the Helsinki Declaration and EU Regulation 2016/679 (GDPR).

A search was conducted by accessing the FPG repository for aggregated and anonymized data, and hospitalizations between 1 January 2019 and 31 October 2021 for malignant uterine cancer surgery were selected according to the National Outcomes Program criteria (25). Among them, those whose discharge hospital form (SDO) reported the ICD9CM codes of at least one surgical procedure on uterus and uterine adnexa and lymph nodes were included in the analysis. Hospitalizations in which the operating session included urinary procedures in addition to uterine surgery were excluded from the analysis, since urinary procedures give rise to different hospitalization trajectories than gynecological surgery alone, both in terms of hospital stays and DRG classification, without taking into account the greater oncological severity of a gynecological tumor that has attacked the urinary tract. Based on ICD9CM codes, the included hospitalizations were subsequently stratified, according to the surgical approach, in laparotomy, laparoscopic, and robotic interventions. For each surgical approach, mean values (with 95% confidence intervals, CIs) were calculated for the following variables: DRG (diagnosis-related group) amount; costs of ordinary inpatient stay, intensive care unit inpatient stay, operating rooms, medical devices, and other healthcare services; and hospitalization's economic margin (i.e., the difference between revenues and costs incurred by the hospital). In the case of robotic surgery, the cost of using the devices took into account the running costs of the robot for the hospital. The ANOVA test was used to assess whether mean values in the three scenarios (laparotomic, laparoscopic, and robotic) were different. In case of statistically significant differences between the three groups detected by the ANOVA test, we proceeded with two-by-two comparisons, to assess differences in means values with the Student's *t*-test: *p*-values ≤ 0.05 were considered statistically significant. Moreover, for each surgical approach, the percentage of readmissions within 30 days (95% CI) from hospital discharge was considered. Differences in 30-day readmissions frequency were assessed through relative risks (RRs) of 30-day readmission, calculated for the three surgical approaches compared to each other (laparoscopic vs. robotic, laparotomy vs. robotic, and laparotomy vs. laparoscopic surgery).

Finally, considering the average costs and revenues of robotic and laparoscopic procedures, a break-even analysis was carried out. The break-even analysis aims to establish a threshold within which costs and revenues for a given output will balance (26). In our case, we evaluated surgical production according to two basic modalities: classic laparoscopy and robot-assisted laparoscopy. These two approaches to the same surgical procedure (and thus to achieve the same output (DRG), for which the hospital receives the same revenue) are expected to have different production costs, especially considering the operating costs of the robot. The economic margin of robotic procedures was then evaluated against the margin of non-robotic procedures to establish sustainability scenarios for the hospital. From the point of view of hospital management, interested in pursuing value and sustainability in healthcare, it evaluated the volume of robotic and non-robotic surgical admissions for which costs and revenues (sum of DRGs) for hospital are equivalent, according to the logic of supply governance.

Thus, the formula used in the break-even analysis was the following equation: Number of robotic procedures × (Revenues of robotic procedure admissions − costs of robotic procedure admissions) − Number of laparoscopic procedures × (Revenues of laparoscopic procedure admissions − costs of laparoscopic procedure admissions) = 0.

Normalizing the number of laparoscopic procedures to the value 1 and considering the margin as the difference between revenues and costs, it is possible to make the equation explicit in these terms: Number of robotic procedures = –(Margin of laparoscopic procedure admissions)/(Margin of robotic procedure admissions). The analysis was conducted on the central mean values and extreme values of the confidence intervals.

Statistical analysis was performed by using STATA software (version 17).

## Results

A total number of 1336 hospitalizations were included in the study, 366 with robotic, 591 with laparoscopic, and 379 with laparotomy surgery. Tables 1, 2, respectively, report descriptive statistics for hospitalizations considered and differences among the three hospitalizations' categories based on the comparison of the three surgical approaches to each other. Hospitalizations with laparotomy surgery had the highest average DRG reimbursement rate (6269.19 €; 95%CI: 6237.26 €−6301.11 €), length of stay (6.24; 95%CI: 5.96–6.53), and length of stay costs (1997.68 €; 95%CI: 1907.14–2088.22 € and 91.82 €; 95%CI: 46.68 €−136.96 € for ordinary inpatient stay and ICU inpatient stay, respectively), other health service costs (331.13 €; 95%CI: 304.02 €−358.24 €), operating rooms costs (2843.35 €; 95%CI: 2769.34 €−2917.36 €), and 30-day readmissions percentage (4%; 95%CI: 2.0%−5.9%).

**Table 1:** Costs and revenues of hospitalizations for malignant uterine cancer surgery: ANOVA.

	**Robotic surgery (n. 366 hospitalizations)**			**Laparoscopic surgery (n. 591 hospitalizations)**			**Laparotomy surgery (n. 379 hospitalizations)**			**ANOVA**
	**Mean**	**CI 95%**		**Mean**	**CI 95%**		**Mean**	**CI 95%**		***p***
DRG reimbursement rate	6,038.63 €	5,972.11 €	6 105.16 €	6,054.07 €	6, 000.06 €	6,108.09 €	6,269.19 €	6,237.26 €	6,301.11 €	< 0.001
Lenght of stay	3.36	3.14	3.58	3.55	3.41	3.70	6.24	5.96	6.53	< 0.001
Ordinary inpatient stay cost	1,074.54 €	1,003.78 €	1,145.29 €	1,136.51 €	1,090.09 €	1,182.94 €	1,997.68 €	1,907.14 €	2,088.22 €	< 0.001
ICU inpatient stay cost	72.13 €	38.89 €	105.37 €	46.70 €	22.77 €	70.63 €	91.82 €	46.68	136.96	0.143
Other health services cost	219.33 €	202.41 €	236.25 €	184.65 €	172.76 €	196.54 €	331.13 €	304.02 €	358.24 €	< 0.001
Operating rooms cost	2,078.51 €	2,023.41 €	2,133.61 €	2,044.07 €	1,992.34 €	2,095.81 €	2,843.35 €	2,769.34 €	2,917.36 €	< 0.001
Medical devices cost	3,549.37 €	3,459.32 €	3,639.43 €	660.02 €	622.53 €	697.52 €	699.44 €	657.47 €	741.40 €	< 0.001
Economic margin	−1,069.18 €	−1,240.44 €	−897.92 €	1,692.21 €	1,531.75 €	1,852.66 €	188.28 €	−10.99 €	387.55 €	< 0.001
30-days readmissions	1.4%	0.2%	2.6%	2.0%	0.9%	3.2%	4.0%	2.0%	5.9%	-

**Table 2:** Differences among the three hospitalizations' categories based on the comparison of the three surgical approaches to each other (Student's *t*-test).

	**Differences between robotic and laparoscopic surgery**				**Differences between robotic and laparotomy surgery**				**Differences between laparoscopic and laparotomy surgery**			
	**Mean**	**CI 95%**		***p***	**Mean**	**CI 95%**		***p***	**Mean**	**CI 95%**		***p***
DRG reimbursement rate	−15.44 €	−101.87 €	71.00 €	*0.726*	−230.55 €	−303.67 €	−157.43 €	*<**0.001***	−215.11 €	−287.34 €	−142.88 €	*<**0.001***
Lenght of stay	−0.19	−0.45	0.06	*0.135*	−2.88	−3.25	−2.52	*<**0.001***	−2.69	−3.01	−2.37	*<**0.001***
Ordinary inpatient stay cost	−61.98 €	−143.19 €	19.24 €	*0.135*	−923.14 €	−1 038.76 €	−807.53 €	*** < 0.001***	−861.16 €	−963.13 €	−759.20 €	*** < 0.001***
ICU inpatient stay cost	25.43 €	−14.73 €	65.59 €	*0.214*	−19.69 €	−76.15 €	36.77 €	*0.494*	−45.12 €	−96.32 €	6.08 €	*0.060*
Other health services cost	34.68 €	14.52 €	54.84 €	*** < 0.001***	−111.80 €	−144.06 €	−79.54 €	*** < 0.001***	−146.48 €	−176.15 €	−116.81 €	*** < 0.001***
Operating rooms cost	34.44 €	−44.42 €	113.29 €	*0.392*	−764.84 €	−857.75 €	−671.93 €	*** < 0.001***	−799.28 €	−889.73 €	−708.83 €	*** < 0.001***
Medical devices cost	2 889.35 €	2 803.79 €	2 974.89 €	*** < 0.001***	2 849.94 €	2 750.35 €	2 949.53 €	*** < 0.001***	−39.41 €	−97.09 €	18.27 €	*0.180*
Economic margin	−2 761.38 €	−3 006.11 €	−2 516.66 €	***< 0.001***	−1 257.46 €	−1 521.41 €	−993.53 €	***< 0.001***	1 503.93 €	1 247.46 €	1 760.39 €	***< 0.001***
30–days readmissions	−0.7%	−2.3%	1.0%	*0.450*	−2.6%	−4.9%	−0.3%	***0.029***	−1.9%	−4.2%	0.3%	*0.075*

The italics and bold values indicated the calculated p-values of the statistic that are statistically significant (*p* ≤ 0.05).

Hospitalizations with robotic surgery had the lowest DRG average reimbursement rate (6038.63 €; 95%CI: 5972.11 €−6105.16 €), length of stay (3.36; 95%CI: 3.14–3.58), average length of stay costs (1074.54 €; 95CI: 1003.78 €−1145.29 € and 72.13 €; 95%CI: 38.89 €−105.37 € for ordinary inpatient stay and ICU inpatient stay, respectively), and 30-day readmissions percentage (1.4%; 95%CI: 0.2%−2.6%) and the highest medical devices average costs (3549.37 €; 95%CI: 3459.32 €−3639.43 €).

Hospitalizations with laparoscopic surgery had the lowest other health service costs (184.65 €; 95%CI: 172.76 €−196.54 €), operating rooms costs (2044.07 €; 95%CI: 1992.34 €−2095.81 €), and medical device costs (660.02 €; 95% CI: 622.53 €−697.52 €) (Table 1).

ANOVA shows significant differences (*p* < 0.001, Table 1) among the averages values of the three approaches (laparotomic, laparoscopic, and robotic), with the exception of the costs of intensive care, which, however, in fact concern a minority of hospitalizations.

Robotic surgery, compared to laparotomy, was characterized by significantly lower DRG reimbursement (*p* < *0.001*), length of stay (*p* < *0.001*), and 30-day readmissions percentage (*p* = *0.029)* and significantly higher medical device costs (*p* < *0.001*). All other cost items were significantly lower *(p* < *0.001)*, except for ICU inpatient stay cost (*p* = *0.494*) (Table 2).

Laparoscopic surgery, compared to laparotomy, showed significantly lower DRG reimbursement (*p* < *0.001*) and length of stay (*p* < *0.001*), but no statistically significant difference for 30-day readmissions percentage (*p* = *0.075*). All cost items were significantly lower, except for ICU inpatient stay (*p* = *0.06*) and medical devices (*p* = *0.18*) (Table 2).

Robotic surgery, compared to laparoscopic surgery, was characterized by a lower DRG remuneration, hospital length of stay, and 30-day readmissions percentage, although without statistically significant differences (*p* = *0.726, p* = *0.135*, and *p* = *0.45*, respectively), and significantly higher device costs and other health service costs (*p* < *0.001*) (Table 2).

Average economic margins were−1069.18 € (95%CI: −1240.44-−897.92 €) for robotic, 1692.21 € (95%CI: 1531.75 €−1852.66 €) for laparoscopic, and 188.28 € (95%CI: −10.99 €−387.55 €) for laparotomy surgery (Table 1). Differences in economic margins of the three approaches compared with each other (robotic vs. laparotomy, laparoscopic vs. laparotomy, and robotic vs. laparoscopic surgery) were all statistically significant (*p* < *0.001*) (Table 2).

RRs of 30-day readmission were 1.49 (95%CI: 0.53–4.18), 2.90 (95%CI: 1.06–7.89), and 1.95 (95%CI: 0.92–4.12) for laparoscopic vs. robotic, laparotomy vs. robotic, and laparotomy vs. laparoscopic surgery, respectively. Laparotomy's 30-day readmission RR was almost three times that of robotic surgery, albeit with a confidence interval bordering on statistical significance (Table 3).

**Table 3:** Relative risk of 30–day readmission of the three surgical approaches compared to each other.

	**RR**	**IC 95%**	
Laparoscopic vs. robotic surgery	1.49	0.53	4.18
Laparotomy vs. robotic surgery	2.90	1.06	7.89
Laparotomy vs. laparoscopic surgery	1.95	0.92	4.12

Regarding the break-even analysis, the comparison of the economic margin (understood as the difference between costs and revenues) of laparoscopic surgery with that of robotic surgery showed that on average, for each malignant uterine cancer elective surgery performed laparoscopically, 1.58 elective robotic surgeries are sustainable for the hospital (95% CI: 1.23–2.06). In fact, admissions with laparoscopic procedures have an average positive margin of about 1690 euros, while admissions with robotic procedures generate a loss of about 1070 euros: It follows that one admission with a laparoscopic procedure theoretically provides the capacity to grant about 1.5 admissions with robotic procedures.

## Discussion

Our results highlight an improvement in terms of patient outcomes, expressed by the 30-day readmissions indicator, when using the robotic surgical technique, compared to both laparoscopic and laparotomic ones. Nevertheless, statistical significance is obtained only when the robotic surgery is compared with laparotomy; statistical significance on the relative risk of readmission is not obtained comparing laparoscopy with laparotomy.

Data show, in terms of duration of hospitalization as well, that the robotic surgery allows a reduction in the parameter vs. both laparoscopic and laparotomic surgeries, but it is statistically significant only when compared to the latter.

The advantages of robotic surgery in terms of outcome identified by our study are in line with the literature. A systematic review and meta-analysis by Ind et al. (21), comparing the robotic and laparoscopic techniques, reported that duration of hospitalization was lower in patients treated with robotic surgery. Other outcomes considered by Ind et al. were blood loss, number of conversions to laparotomy, and overall complications, all of which were lower in patients treated with the robotic technique (21).

Similarly, the retrospective study by Casarin et al. (19), analyzing data from hospitals in the United States between 2008 and 2015 inherent to hysterectomies in adult patients, supported the finding that the robotic surgery results in a shorter hospitalization when compared to the laparoscopic and laparotomic techniques. Moreover, the study showed a lower 30-day complications rate for robotic surgery compared with laparotomy. In terms of 30-day readmissions, it reported data confirming our observations, with a lower rate for robotic surgery compared to laparotomy (19).

Regarding the evaluation of costs, our analysis shows that robotic surgery admission has lower costs than laparotomic technique in several parameters, assuming statistical significance in case of costs related to the ordinary inpatient stay, other health services, and operating room.

On the contrary, the expenses incurred with robotic surgery in terms of medical devices are significantly higher than the costs associated with laparotomic technique. These data result in a negative economic margin of robotic surgery in comparison with laparotomy. In addition, it can be seen that economic revenue for admission with robotic surgery is statistically significantly lower than revenue for admission with laparotomic surgery. This is certainly attributable to the higher complexity of patients for whom open surgery is required, as they are not suitable for robotic or laparoscopic procedures. A higher complexity of patients in fact generates a higher reimbursement for the hospital, in accordance with the logic of DRG reimbursement (27) (in our sample, it was found that laparotomy surgery is more often associated with a diagnosis of ovarian malignancy, generating a DRG with a higher economic amount).

Compared to the laparoscopic technique, however, the robotic technique has a reduced ordinary inpatient stay cost, although not statistically significant, which presents higher costs related to ICU inpatient stay, operating room, other health services, and medical devices, with statistical significance achieved for the last two only. The difference in the economic value of medical devices, considering the use of the robot and its management costs, is huge, representing almost the whole difference in the economic margin, especially in light of the fact that the value of the DRG amount is not significantly different in the comparison between robotics and laparoscopic surgery. It can be seen that the average difference in the value of medical devices in the two approaches is around € 2,890; the average difference in the value of the operating margin is about € 2,760, showing how all the economic loss in the comparison between robotic and laparoscopic surgery is precisely attributable to the management costs of the technologies used in the operating room. In view of this, it is also useful to underline that the DRG rates in force in Italy do not provide for specific reimbursements related to surgical approaches, except in some cases (e.g., laparoscopic cholecystectomy) (27), reimbursing hospitalizations according to a classification which almost always disregards the technology used.

The DRG system derives from the research on the hospital production function started in 1967 by the group of Yale University, in the United States, coordinated by Robert Fetter (28, 29).

The DRG classification system is a method of categorizing patients for health insurance purposes, to control costs and facilitate reimbursement by third-party providers for the use of medical services and equipment. Using the DRG system, patients are classified, according to a number of variables, into a limited number of groups to form clinically meaningful, but relatively homogeneous, patterns of resource consumption (30).

In Italy, an initial version of the Medicare DRGs was used from the 1st of January 1995 to the end of 2005. A subsequent version of the Medicare DRGs was used from the beginning of 2006 to the end of 2008. The current version of the Medicare DRGs, finally, has been in use since the beginning of 2009; therefore, in our country, there is a delay in updating the DRGs (31). The process of obsolescence toward which DRG are heading partly justifies the economically disadvantageous margins that robotic surgery suffers in terms of reimbursement. This is an example of healthcare payment systems failing to keep pace with the technological advances in modern medicine (32).

Concerning our findings, although at a first analysis of our data the robotic technique does not seem to ensure concrete economic advantages when compared to the other two techniques, it is necessary to consider the relatively recent introduction of robotic surgery in our University Hospital.

Our study shows that even now, the costs of the operating room with robotic surgery, one of the items with greater economic weight, are significantly lower than the laparotomic technique and slightly lower than the laparoscopic technique: We expect that, as a consequence of the learning curve, an improvement in terms of skill by our surgeons will lead to a reduction in operating time and a consequent reduction in operating room costs.

Exemplary in this regard is the work of Avonstondt et al. in 2017, in which differences between the costs of the robotic technique on its introduction and 5 years later were measured: The results were unequivocal, with a reduction in mean total costs and mean operative costs. The reduction in mean operative costs was given principally by the reduction of anesthesia and mean operating room costs. At once, they reported a reduction in mean procedure time and mean operative time, showing that the decrease in costs was mainly due to reduced operative times (24).

In addition to considerations about the effectiveness of robotic surgery, it is also necessary to take into account the value this technique assumes within a valuable context such as the University Hospital analyzed by our study (33, 34).

This is from the point of view of both the unique gynecological oncology's activity volumes of the hospital in the Italian panorama and the relevant academic value inherent in the practice of robotic surgery (25, 35).

Based on these considerations, and in light of the findings of our study, we can say that, on the one hand, it is certainly neither feasible nor appropriate to preclude the use of robotic surgery in the context examined, but, on the other, it is necessary to search for an effective clinical governance tool to distribute surgical volumes between laparotomic, laparoscopic, and robotic procedures, to ensure the sustainability of malignant uterine cancer surgery in a value-based perspective.

The methodology we used to address this issue is the break-even analysis. It consists of the study of the interrelationships between costs, sales volume, and prices of a business/service/product with the objective of identifying the break-even point. The last is often the time at which the fixed and variable costs involved in the production and distribution of a product are matched by its overall sales; generally, that is the point at which total costs are exactly equal to revenues (26).

The results of the analysis show how, by comparing the economic margin, the break-even point, and consequently the suggested ratio between robotic and laparoscopic surgery, is reached in the value of 1.58. Therefore, about up to 1.5 robotic procedures could be performed for every laparoscopic procedure (three robotic for every two laparoscopic procedures), to make robotic surgery sustainable, safeguarding the economic equilibrium alongside the improvement of health outcomes in the logic of value.

The use of the break-even approach allows to promote the value-based view by identifying a useful criterion for the planning of interventions for uterine malignancies, all of this while ensuring the use of robotic surgery, with its advantages both in terms of surgeon learning curve and clinical outcomes, and the sustainability of the system. However, it is worth pointing out that, as in any break-even analysis, the “zero point” or break-even point depends strictly on how accurately it could be the calculation of revenues and costs, both fixed and variable, for the hospital. In our case, while for revenues we can easily refer to DRG payment system and for variable costs to production factors such as inpatient stays, devices, and operating room occupancy, specific to selected admissions, fixed costs are not immediately reversible on same admissions, for which DRGs have theoretically to pay both fixed and variable costs. In this perspective, one limitation of our break-even analysis is that it essentially concerns living costs per performance output. Our study is not free from some limitations indeed, among which first of all the fact that it is not a multicenter study, an aspect mitigated by the large activity volumes of our hospital in terms of malignant uterine cancer surgery (25). Second, the only outcome indicator used is that of readmissions within 30 days from hospital discharge, data borrowed from administrative sources.

Among strengths, as previously mentioned, there is the large number of cases treated in our hospital which is a reference center at the national level for oncological surgery of the uterus, and the accurate methodology was adopted.

Our study opens up a number of future implications both in terms of healthcare management and research. As for healthcare management, it made it possible to identify, in the context considered, the break-even analysis as a useful tool to support the planning and governance of malignant uterine cancer surgery activities. The systematic application of this tool will allow defining over time the right distribution of robotic, laparoscopic, and laparotomy surgeries' volumes to perform to ensure both quality and economic-financial balance and therefore value of uterine oncological surgery in our University Hospital. Concerning research, this study paves the way for a multicentric study, the extension of outcomes of malignant uterine surgery to be considered and assessed, and the future inclusion of other therapeutic interventions in the analysis.

## Data availability statement

The raw data supporting the conclusions of this article will be made available by the authors, without undue reservation.

## Author contributions

MS, GA, and AD: conception and design of the study, acquisition, analysis, interpretation of data, drafting the article, and revising the article critically for intellectual content. EL and TO: analysis and interpretation of data and drafting the article. PL: analysis and interpretation of data and revising the article critically for intellectual content. GS and RDAB: conception of the study and critical revision of the article for intellectual content. All authors contributed to the article and approved the submitted version.

## Funding

This research project and its publication was funded by Università Cattolica del Sacro Cuore.

## Conflict of interest

The authors declare that the research was conducted in the absence of any commercial or financial relationships that could be construed as a potential conflict of interest.

## Publisher's note

All claims expressed in this article are solely those of the authors and do not necessarily represent those of their affiliated organizations, or those of the publisher, the editors and the reviewers. Any product that may be evaluated in this article, or claim that may be made by its manufacturer, is not guaranteed or endorsed by the publisher.
